# What matters in making demand-based decisions: Time alone or difficulty too?

**DOI:** 10.1007/s00426-021-01583-6

**Published:** 2021-09-21

**Authors:** Markus Janczyk, Iman Feghhi, David A. Rosenbaum

**Affiliations:** 1 Department of Psychology, University of Bremen, Bremen, Germany; 2 Department of Psychology, UC Riverside, Riverside, CA USA

## Abstract

Which task is easier, doing arithmetic problems of specified form for some specified duration, or carrying a bucket of specified weight over some specified distance? If it is possible to choose between the “more cognitive” task and the “more physical” task, how are the difficulty levels of the tasks compared? We conducted two experiments in which participants chose the easier of two tasks, one that involved solving addition or multiplication problems (Experiment 1) or addition problems with different numbers of addends (Experiment 2) for varying amounts of time (in both experiments), and one that involved carrying a bucket of different weights over a fixed distance (in both experiments). We found that the probability of choosing to do the bucket task was higher when the bucket was empty than when it was weighted, and increased when the cognitive task was harder and its duration grew. We could account for the choice probabilities by mapping the independent variables onto one abstract variable, Φ. The functional identity of Φ remains to be determined. It could be interpreted as an inferred effort variable, subjective duration, or an abstract, amodal common code for difficulty.

## Introduction

Decisions between courses of actions are often assumed to be driven by attempts to minimize effort (Dunn & Risko, 2019; Dunn et al., 2016; Fournier et al., 2019; Gray et al., 2006; Hull, 1943; Kool et al., 2010; Zipf, 1949). What effort actually is, however, is unclear. Some researchers have suggested that effort amounts to time (Gray & Fu, 2004; Gray et al., 2006). Others have argued that effort amounts to demands on executive control (e.g., Kool et al., 2010; Taatgen, 2007), often operationalized with regard to task switching (Rogers & Monsell, 1995). Yet another hypothesis is that effort amounts to error avoidance or the associated demands (Dunn et al., 2019a, 2019b). A priori, any of these factors could hold in different circumstances, but it would be more satisfying to develop an understanding of the effort and task choice that appeals to some deeper, less ad hoc, understanding.

The main method for studying the effect of effort on choices is the Demand Selection Task (DST) used by Dunn and Risko (2019), Botvinick and Rosen (2009), and Kool et al. (2010), along with others who used the same method without explicitly referring to it by that name (Rosenbaum et al., 2013).^1^ The DST has been used in investigations of the effects of the factors mentioned above, that is, the effect of task switching, time, and error likelihood, but has also been used in evaluations of physiological (Botvinick & Rosen, 2009) and neurological (McGuire & Botvinick, 2010) consequences of task engagement or anticipation. This body of work has shown that it is not necessarily the *objective* costs (of whatever kind) posed by a task, but rather “subjective costs and some degree of explicit awareness of said costs” (Dunn et al., 2016, p. 1373) that drive effort-based decision-making (see also Dunn et al., 2019a, 2019b; Gold et al., 2015). Accordingly, Dunn et al. (2016) have suggested that multiple cues can contribute to the evaluation of effort, provided those cues are sufficiently salient to be noticed (Dunn & Risko, 2019).

In typical DSTs, the choice options vary on cognitive dimensions. However, the same method can be used to investigate the comparison of physical and mental tasks (Feghhi & Rosenbaum, 2019, 2021; Feghhi et al., 2021; Potts et al., 2018; Rosenbaum & Bui, 2019; Rosenbaum et al., 2013). For example, if one chooses between doing ten challenging math problems or moving a wheelbarrow full of rocks back and forth between a couple of locations ten times, what would one choose? On what basis would effort be compared? The choice would surely depend on features of the tasks—how challenging the math problems were, how many of them there were, how far apart the rock locations were, how heavy the rocks were, how many trips were necessary, and so on. That these factors would affect the choice suggests that different kinds of effort can be compared. If so, what are the core elements for the decisions?

The tasks mentioned above may be said to differentially tax “brain” and “brawn.” One task, doing math problems, is “more cognitive.” The other, moving rocks, is “more physical.” Of course, these terms are intuitive at best, for “mental tasks” also require physical enactment, and “physical tasks” also require thought. If physical tasks only required brawn but no brain, robots would be more capable than they are of complex actions in unpredictable environments, and doing physical tasks would not affect cognitive performance or vice versa, though such interactions have been observed (e.g., Weigelt et al., 2009; Zhang et al., 2014).

As just intimated, the question posed here is, what aspect(s) of such tasks contribute(s) to a “common currency” that may be used to compare the effort associated with different kinds of tasks and, for that matter, tasks of a given kind? That there might be a common currency is suggested by the fact that when people compare the difficulty of physical and mental tasks—not math and rock-transport tasks, as in the just mentioned example, but digit memorization and walking through gaps of varying width—their choices are systematic (Feghhi & Rosenbaum, 2019, 2021; Feghhi et al., 2021). Even so, it is possible that the subjective difficulty of each task is measured with a *different* metric. For example, attentional demands might be used to determine mental difficulty, whereas caloric consumption might be used to determine physical difficulty. If mental difficulty values and physical difficulty values were mapped onto one another—say from smallest to largest in both cases—it might be possible to decide between the two kinds of tasks based on the relative positions of their values. No common currency would be needed.

Notwithstanding the latter possibility, several common-currency candidates have been considered in the literature. Energy has been suggested (Craig, 2013; Job et al., 2010), though, as far as we can tell, that term has been used metaphorically rather than literally as defined in physics (e.g., Navon & Miller, 2002; Tombu & Jolicoeur, 2003). Likelihood of error may also be considered (Dunn et al., 2019a, 2019b), but Feghhi and Rosenbaum (2019, 2021) showed that error reduction is not the *sine qua non* of the task choice. The latter point can be reached by recognizing that the cost of a car crash is much higher than the cost of a math-homework mistake even though the probabilities of the two events might be the same. Finally, time on task has been considered as well (Gray et al., 2006; Potts et al., 2018; Rosenbaum & Bui, 2019), and this candidate will be a focus of the current investigation.

### (Subjective) time and choice

One of the studies just cited, Potts et al. (2018), served as the basis for the two experiments reported here. In the Potts et al. (2018) study, participants chose between a cognitive task (counting up to target values of 8, 12, 16, or 20) and a physical task (picking up a bucket from a stool and carrying it to a target stool). Two stools stood at the end of an alley, and four other stools stood midway from a starting position to the target stools, with two stools each to the left and to the right (for an illustration, see Fig. 1 in Potts et al., 2018). Whether the bucket was on the left or right and whether it required a short or long reach was varied within-participants, as were the target values for the counting task. The combinations of the four-count values and four bucket positions resulted in 16 trials per participant. There was also a between-participants factor with three levels: the bucket was (1) empty, (2) filled with 3.5 pounds of pennies, or (3) filled with 7 pounds of pennies.

**Fig. 1:**
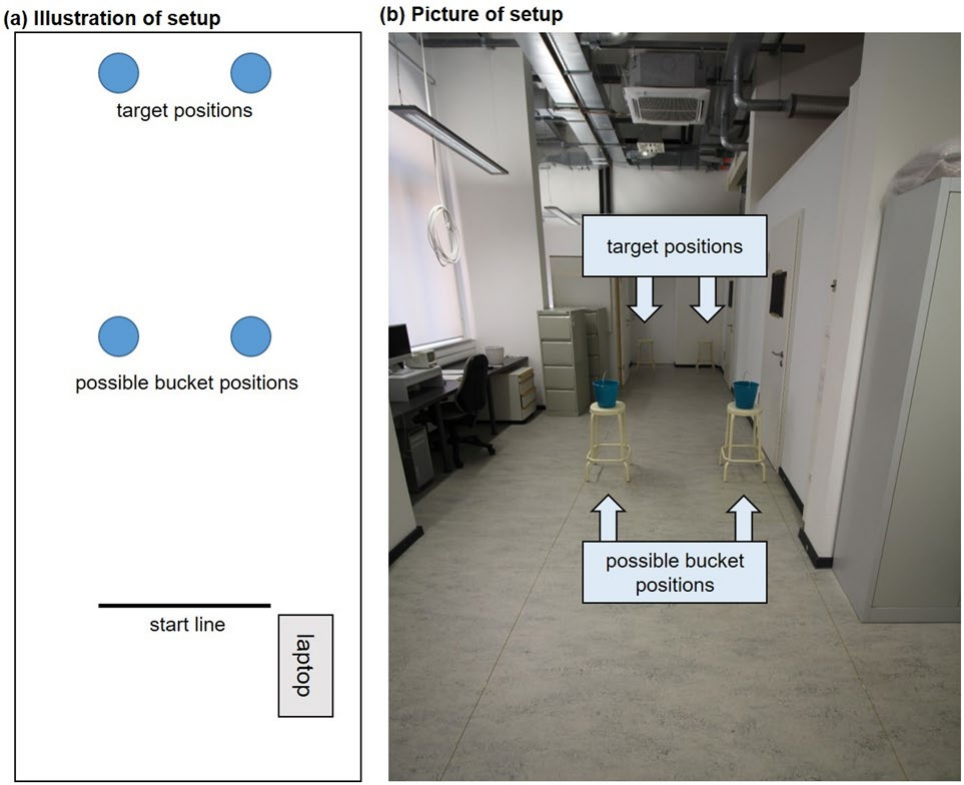
Illustration of the experimental setup. **a** Overhead sketch. **b** Photograph of the real setup with the bucket on both stools for illustration only. In the experiment, only one bucket was present, either on the left or right

**Fig. 2:**
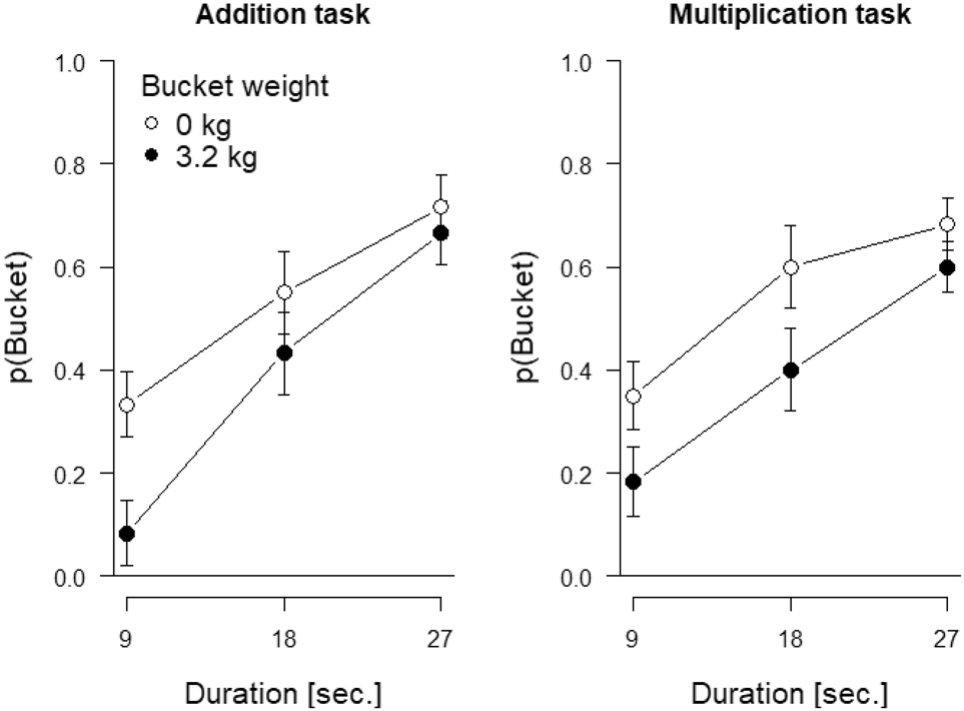
Probability of choosing the bucket, *p*(Bucket), in Experiment 1, as a function of the duration of the cognitive task (x-axis), bucket weight (separate lines), and difficulty of the cognitive task (i.e., addition vs. multiplication) as separate panels. Error bars show 95% within-participants confidence intervals for the difference between the empty and the loaded bucket, calculated separately for each cognitive task difficulty and duration

Potts et al. (2018) observed that the probability of choosing the bucket, *p*(Bucket), rather than the counting task increased with the count targets and was larger for short reaches than for long reaches (see Fig. 2 in that study).^2^ In contrast, bucket side or bucket weight had no effect on the choices. Most important was the reliable effect of task-completion time: Chosen tasks took less time than unchosen tasks. The authors also observed that the choice probabilities could be better fitted if *subjective* time rather than *objective* time was input into a model. The subjective time model that Potts et al. developed ascribed 5 extra seconds of subjectively experienced time to long-reach tasks compared to short-reach tasks. The time of 5 extra seconds was found to maximize the goodness of fit of the model to the data.^3^

To test the hypothesis that subjective time was the basis for their *p*(Bucket) data, Potts et al. (2018) ran a second experiment. Here, they repeated the first experiment, though with empty buckets only, and replicated the results of Experiment 1. Yet, in addition to collecting choice and performance data, they asked a new group of participants to estimate how long they would spend on each of the tasks. These time estimates (see Fig. 6 in that study) were longer for long-reach tasks than for short-reach tasks by a wider margin than the difference in objective times for the long and short reaches. This outcome is in line with the model-fitting that Potts et al. (2018) did. Participants’ time estimates for the counting tasks also differed from the objective counting times. Interestingly, the time estimates exceeded the objective counting times by an amount that grew as the count maximum (the target value at which counting could stop) increased. When the Experiment 2 *p*(Bucket) data were fitted with the obtained subjective times, the fit was better than when objective times were used.

### The present study

In the present study, we conducted two experiments following up the work by Potts et al. (2018). We embarked on these new experiments because there were confounds in the earlier study, which—though acknowledged by the authors—were not tracked down by them. Specifically, while time for counting increased with larger target values, counting to higher target values could have taxed resources and executive control requirements in ways that happened only incidentally to be indexed by time. In addition, counting to higher target values could have led to more errors. Participants’ decisions could have been driven by any of these factors. We sought to find out which factor(s) really mattered because, as indicated in the title of this article, we wanted to know whether time or difficulty is the principal basis for choosing actions of the kinds investigated here.

This question was especially important given the earlier influential report by Kool et al. (2010), who reported that for a (purely cognitive) DST, time on task was not the primary basis for determining effort, nor were error rates or, conversely, rates of accumulation of positive feedback. What mattered, the authors concluded, were the requirements imposed on executive control, for example, operationalized by different percentages of task switches. Because Potts et al. (2018) reported that subjective time better accounted for task choices than objective time, one could argue that participants in the study by Kool et al. actually formed estimates of task completion times and relied on those psychologically mediated times to make their choices.^4^

In the experiments reported here, the participants were asked to choose the easier of two tasks, one being a cognitive task, the other a physical task, as in the study of Potts et al. (2018). For the cognitive task, they were confronted with math problems—either adding or multiplying two one-digit numbers in Experiment 1 or adding and subtracting 2 or 4 one-digit numbers in Experiment 2—for a specified duration that was disclosed at the start of each trial. This allowed us to assess the (possible separate) contributions of cognitive difficulty and time requirements. The way the math time requirement was implemented was to allow participants to complete the last problem presented to them before the computer-controlled deadline was up.^5^ This procedure differed from the one used by Potts et al. (2018) where participants could, in principle, modulate the duration of their counting by varying their counting rates.

For the physical task, participants were asked to carry a bucket to the end of the alley. Regarding this task, we varied bucket weight in a way that Potts et al. (2018) did not. In the first experiment of Potts et al. (2018), bucket weight was varied as a between-participants factor. Potts et al. (2018) failed to find an effect of bucket weight on *p*(Bucket), so they did not vary bucket weight in their second experiment, where the other (more positive) innovation was to obtain subjective time estimates. Rather, the bucket was empty in Potts et al.’s (2018) second experiment. The lack of an effect of bucket weight in Experiment 1 was unexpected, as the authors noted; yet, its absence could have been due to the fact that this factor was varied between-participants (see also Birnbaum, 1999; Schweitzer et al., 2013). In the present experiments, we varied bucket weight within-participants to see if we would pick up an effect when variations in that factor became more salient. In other respects, the design and method used here were meant to simulate those of Potts et al. (2018).

In sum, in the present work, we sought to extend the study by Potts et al. (2018) by disentangling the effects of mere time requirements to perform a cognitive task (math problems), the difficulty of the cognitive task, and the difficulty of a physical task (bucket carrying) on task choice.

## Experiment 1

### Method

#### Participants

Thirty people (mean age = 25.1 years, 19 female) from the Tübingen area (Germany) participated for money or course credit. A power analysis suggested that this sample size was large enough to detect an effect of size *d* ≥ 0.53 with a power of 1 − *β* = 0.80 (two-sided paired *t* test, *α* = 0.05). All participants reported normal or corrected-to-normal vision, were naive to the hypotheses, and signed an informed consent form prior to data collection. Data were collected after participants completed an unrelated experiment.

#### Apparatus and stimuli

The setup for the experiment is shown in Fig. 1. In the *physical (bucket) task*, four stools (height: 75 cm) were used as platforms (30 cm diameter). Two were closer to the participants’ start position and two were farther away. A bucket was placed on one of the closer stools at the start of each trial. One bucket was blue and the other was grey. One of the buckets was empty (0.0 kg). The other was filled with gravel (3.2 kg). Participants were informed of the color-weight mapping (which was counterbalanced across participants) that applied to them and were given a chance before the main experiment began to heft each bucket to get a clear haptic sense of their weights. The buckets’ handles were fixed in an upright position to facilitate easy grasping and were oriented parallel to the long axis of the walkway. Whereas both panels of Fig. 1 show both possible bucket positions, in the actual experiment only one bucket was presented per trial, either on the left or right. The distance from the start line to the bucket positions was always 376 cm. The distance from the start line to the target positions was always 750 cm. The alley was always 90 cm wide.

The *cognitive task* was administered on a laptop, which was placed to the right of the participant (as in Potts et al., 2018; see Fig. 1a) on a table 100 cm high. The cognitive task was either to add or multiply two digits per trial. We expected multiplication to be judged more difficult than addition (Ashcraft & Guillaume, 2009). In each trial, the participant typed his or her answer, and after hitting the enter button, was shown the next equation. Feedback about accuracy was not provided.

#### Tasks and procedure

At the start of each trial, the participant stood behind the starting line, facing away from the area with the stools and bucket, with eyes closed. At this time, the experimenter prepared the upcoming trial and then told the participant to turn around and look at the laptop. The screen informed the participant about the relevant cognitive task for the upcoming trial (i.e., whether they would add or to multiply digits), and also about its duration, should they choose that task. We provided the participant with two options and asked him or her to choose (and subsequently) perform the easier of the two options, as in the study of Potts et al. (2018). Here, the choice was indicated by pressing the left CTRL key for the bucket task or the right CTRL key for the cognitive task. Depending on the condition, the cognitive task was either addition or multiplication and was to be done for 9, 18, or 27 s. Pilot work showed that 18 s was the approximate time to walk in a normal pace from the start line, pick up a bucket, place it on the target stool, and return to the start line. The other values were chosen to be shorter and longer than this time by equivalent amounts (± 9 s). If participants opted for the bucket task, they were to embark on the task immediately after hitting the corresponding button on the laptop. That way, the laptop button-press served as a proxy for the start time of the chosen act. The proxy for the end time of the bucket task was when the experimenter hit a button at the moment the participant returned to the starting line. If the participant chose the cognitive task, the first equation appeared immediately. After the participant typed in the sum or product for the problem at hand, their act of pressing the Enter key brought up the next equation unless the duration of 9, 18, or 27 s had elapsed.

The entire task had 24 trials based on the combination of 2 bucket locations (left vs. right) × 2 bucket weights (0.0 vs. 3.2 kg) × 2 levels of cognitive task difficulty (addition vs. multiplication) × 3 durations of the cognitive task (9 vs. 18 vs. 27 s).

#### Design and analyses

In an attempt to assess cognitive task difficulty, we compared (1) the number of performed calculations between the addition and multiplication tasks and (2) the error rates in both tasks. In both cases, we averaged over the three durations of the cognitive task. A more difficult task would then be indicated by a smaller number of performed calculations and/or more errors. The main analysis assessed the probability of choosing the bucket, *p*(Bucket), via a repeated measures ANOVA whose independent variables were duration of the cognitive task, difficulty of the cognitive task, and difficulty of the physical task (i.e., bucket weight). Because bucket location had no effect on choices, we aggregated choices regardless of where the bucket stood.^6^

## Results

On average, participants managed to perform 8.46 calculations of the addition task but only 7.47 calculations of the multiplication task, *t*(29) = 2.87, *p* = 0.008, *d* = 0.52. Error percentages were 10.92 and 11.17 in the addition and multiplication task, respectively. This difference was not significant, *t*(29) = 0.04, *p* = 0.966, *d* = 0.01 The probability, *p*(Bucket), of choosing the bucket as a function of the difficulty and duration of the cognitive task and bucket weight is shown in Fig. 2. The impression from the figure is that participants chose the bucket task more often when the bucket was empty than when it was weighted and more often as the duration of the other, cognitive, task increased. Difficulty of the cognitive task (addition versus multiplication) did not have a major impact on bucket choices, as confirmed in the ANOVA for these data (see Table 1 for the complete results). Only the two main effects of cognitive task duration and bucket weight were significant, *F*s ≥ 20.44, *p*s < 0.001. All other effects were non-significant, all *F*s ≤ 1.99, all *p*s ≥ 0.147.

**Table 1:** Statistics of the full three-way ANOVA for Experiment 1

Effect	*F*	*P*	η_p_^2^
Duration	22.08	< .001	0.43
Difficulty	0.01	0.923	< 0.01
Weight	20.44	< 0.001	0.41
Duration × Difficulty	1.28	0.285	0.04
Duration × Weight	1.99	0.147	0.06
Difficulty × Weight	0.07	0.791	< 0.01
Duration × Difficulty × Weight	1.01	0.369	0.03

Difficulty and duration refer to the cognitive task while weight refers to the physical task

## Discussion

Experiment 1 replicated and extended the results of Potts et al. (2018) by showing that when the cognitive task duration was controlled, the longer the cognitive task was to be performed the more often participants chose the alternative bucket task. We also observed a clear effect of bucket weight, that is, physical task difficulty. Participants chose the cognitive task more often when the bucket was loaded than when it was empty. This outcome accords with our expectation that with a within-participants manipulation, where the same participants got physical tasks with varying demands (buckets with different loads), they would show greater sensitivity to the demand levels than was the case in the study of Potts et al. where bucket weight was varied between- participants in their Experiment 1.

Unexpectedly, in the present experiment, the (presumed) difficulty of the cognitive task did not affect choices. Whether the cognitive task was addition or multiplication, it did not matter. Interestingly, the tasks differed in the rate at which problems were solved—more addition problems were solved per time unit than were multiplication problems—but error rates were comparable. Other studies have also reported a lack of differences in error rates between addition and multiplication of two digits, the number of digits per problem used here (e.g., Zhou et al., 2007). However, even though this dissociation may be interesting in itself, the absence of an error-rate difference also suggests that (a) the completion rate was not the determinant of the (perceived) task difficulty and (b) the intended manipulation of cognitive task difficulty was unsuccessful, or at least not strong enough to influence the choices made by our participants. To address this issue, we manipulated cognitive difficulty in a different way in Experiment 2.

## Experiment 2

Most aspects of this experiment were the same as in Experiment 1 with the major change relating to the cognitive task. Because it was unclear whether multiplication was substantially more difficult (objectively and subjectively) than addition, we turned to a different task.

### Method

#### Participants

Forty-eight people (mean age = 23.8 years, 34 female, 15 male) from the Tübingen area (Germany) participated in this experiment for the same criteria as described in Experiment 1. A power analysis with the same parameters as for Experiment 1 indicated that this sample size was sufficient to detect effects of *d* ≥ 0.42.

#### Apparatus, stimuli, task, procedure, design, and analyses

We used the same material and setup for the *physical (bucket) task* as in Experiment 1. The *cognitive task* was changed so that participants were presented with equations involving addition and subtraction of single-digit numbers. Depending on task difficulty, either 1 or 3 successive additions/subtractions were used in an equation (i.e., either 2 or 4 digits occurred on the left side of the equation). For the less difficult condition, the equations were of the form A − B = Z, which we called the 2-digit condition. For the more difficult condition, the equations were of the form A − B + C − D = Z, which we called the 4-digit condition. A solution to each equation was given (on the right side of each equation) and the participants’ task was to decide whether the provided answer was correct or incorrect. In the former case, they were to press the right CTRL key; in the latter case, they were to press the left CTRL key. Whether the shown answer was correct or incorrect was randomly determined per trial. If the shown answer was incorrect, the displayed result differed from the correct result by + 1 or − 1, which was determined randomly.

To ensure that all participants had a clear idea of each task, they started with an exposure period of the cognitive task prior to the main experiment. Both levels (2-digit and 4-digit conditions) were administered 40 times, with the order counterbalanced across participants. After this, the main experiment was conducted in the same manner as described for Experiment 1. For the cognitive task, a new equation appeared after participants pressed the response key each time the duration was still in effect.

The task consisted of 24 trials resulting from the combination of 2 bucket locations (left vs. right) × 2 bucket weights (0.0 vs. 3.2 kg) × 2 levels of the cognitive task difficulty (2 digits vs. 4 digits) × 3 cognitive task durations (9 vs. 18 vs. 27 s). Error rates and RTs during the exposure period were analyzed as a function of cognitive task difficulty (2 vs. 4 digits) to assess task difficulty objectively. The data analysis protocol followed that of Experiment 1.

## Results

In the exposure block, participants made fewer errors in the 2-digit condition than in the 4-digit condition, (5.68 vs. 11.61%), *t*(47) = 5.59, *p* < 0.001, *d* = 0.81, and responses were made more quickly in the 2-digit condition than in the 4-term condition (1851 vs. 5774 ms), *t*(47) = 20.05, *p* < 0.001, *d* = 2.89. These outcomes suggest, as expected, that the 2-digit condition would be less difficult than the 4-digit condition.

**Fig. 3:**
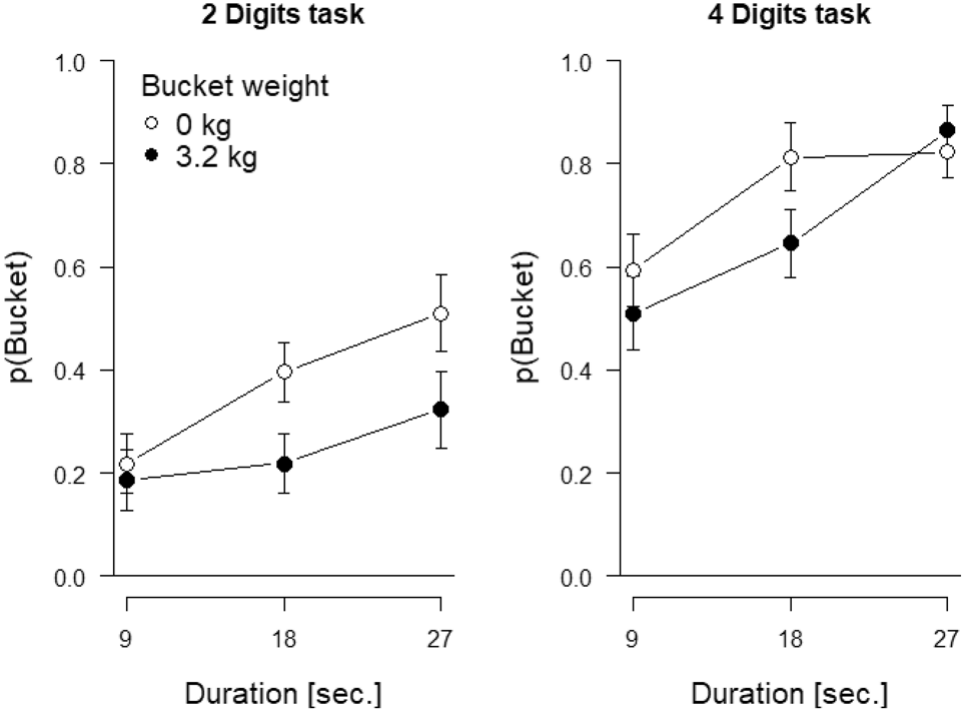
Probability of choosing the bucket task, *p*(Bucket), in Experiment 2 as a function of the duration of the cognitive task (x-axis), bucket weight (separate lines), and difficulty of the cognitive task (2 digits vs. 4 digits) as separate panels. Error bars are 95% within-participants confidence intervals for the difference between the empty and loaded bucket, calculated separately for each cognitive task difficulty and duration

Figure 3 shows the probability of choosing the bucket, *p*(Bucket), as a function of the difficulty and duration of the cognitive task and bucket weight. The impression is that participants chose the bucket task more often as the cognitive task duration increased, when the bucket was empty compared to when the bucket was weighted, and when the cognitive task involved 4 digits compared to 2.

As shown in Table 2, this impression was corroborated by the respective three main effects, all of them being significant, assuming α = 0.05. Because the three-way interaction had a *p*-value that, in traditional hypothesis-testing terms would be “just significant,” we analyzed the two levels of cognitive task difficulty separately with ANOVAs that only had cognitive task duration and bucket weight as repeated measures.

**Table 2:** Statistics of the full three-way ANOVA for Experiment 2

Effect	*F*	*p*	η_p_^2^
Duration	21.13	< 0.001	0.31
Difficulty	89.32	< 0.001	0.66
Weight	9.36	0.004	0.17
Duration × Difficulty	0.87	0.422	0.02
Duration × Weight	2.12	0.126	0.04
Difficulty × Weight	2.04	0.159	0.04
Duration × Difficulty × Weight	3.08	0.050	0.06

Difficulty and duration refer to the cognitive task while weight refers to the physical task

For the 2-digit task, the main effect of duration was significant, *F*(2,94) = 7.36, *p* = 0.001, η_p_^2^ = 0.14, and this was also true for the main effect of bucket weight, *F*(1,47) = 11.41, *p* = 0.001, η_p_^2^ = 0.20. However, the interaction was not significant, *F*(2,94) = 2.03, *p* = 0.137, η_p_^2^ = 0.04. For the 4-digit task, the main effect of duration was significant, *F*(2,94) = 20.29, *p* < 0.001, η_p_^2^ = 0.30, whereas the main effect of weight was not, *F*(1,47) = 3.02, *p* = 0.089, η_p_^2^ = 0.06, and the *p*-value of the interaction fell just below the traditional α value for significance, *F*(2,94) = 3.21, *p* = 0.045, η_p_^2^ = 0.06.^7^

## Discussion

Experiment 2 was similar to Experiment 1 except that we varied the number of digits (2 or 4) in mixed addition and subtraction problems to manipulate the difficulty of the cognitive task. This manipulation differentially taxed participants in ways that the use of addition versus multiplication did not in Experiment 1. Given this outcome, the following conclusions could be drawn. First, there was an effect of the duration of the cognitive task on *p*(Bucket). The longer that duration, the higher the value of *p*(Bucket), replicating what was observed in Experiment 1. Second, loaded buckets were chosen less often than empty ones, also replicating what was observed in Experiment 1, and pointing to an effect of physical task difficulty. Third and finally, there was a clear effect of cognitive task difficulty on the likelihood of choosing the bucket in this experiment. When only two digits would be dealt with, *p*(Bucket) was lower than when four digits would be dealt with. These results show that all three variables—time, cognitive task difficulty, and physical task difficulty—affected choices. Perhaps most important given the main question of this study, the decisions about which task to carry out were not just based on time, as also suggested by Kool et al. (2010).

## General discussion

In this article, we have reported two experiments on choosing between a “more cognitive” task and a “more physical” task. Earlier studies (Potts et al., 2018; Rosenbaum & Bui, 2019) suggested that time may be the underlying metric for choosing a (subjectively) less difficult task. The present study built on those results and aimed to disentangle the contributions of duration and difficulty of a cognitive task—variables that were confounded in the earlier studies—and the difficulty of a physical task. To this end, we employed two cognitive tasks per experiment to manipulate (cognitive) difficulty. In Experiment 1, the cognitive tasks were addition versus multiplication of 2 digits. In Experiment 2, the cognitive tasks were mixed addition and subtraction of 2 versus 4 digits. We also varied how long the tasks were to be performed in both experiments. The durations were 9, 18, or 27 s. These values were based on 18 s as the approximate time of the bucket task, with 9 s and 27 s being 9 s shorter and longer, respectively, than that time. Further, we manipulated the difficulty of the physical task by varying the weight of the bucket, because this variable had unclear effects in earlier studies, where bucket weight was manipulated between-participants. Here we made bucket weight a within-participants factor and crossed this variable with the two levels of cognitive task difficulty and duration for each single participant.

Two results were clear in both experiments. First, the longer the duration of the cognitive task, the more often participants chose the bucket task. Second, when the bucket was loaded, the bucket task was chosen less often than when the bucket was empty. This latter result contrasts with Potts et al.’s (2018) report that bucket weight did not matter, though that study used a between-participants variation for bucket weight; for similar observations and a discussion, see Birnbaum (1999) and Schweitzer et al. (2013). The latter observation may also be discussed in reference to the General Evaluability Theory (GET; Hsee & Zhang, 2010). Varying bucket weight between- versus within-participants can be conceived as implementing a single versus a joint evaluation mode. However, in a single evaluation mode, some reference information is required to allow choices. Dunn et al. (2017) investigated the evaluability of stimulus rotation, set size, and weight. Stimulus rotation appeared as a not evaluable feature and the authors suggested that error or failure knowledge might be required to make a feature evaluable. In our case, bucket weight would probably matter in a between-participants design where bucket weights were increased enough to strain ethical guidelines regarding the loads that one group could be asked to carry. Thus, while there clearly is a point of failure in carrying a weighted bucket, the employed loads might have been too low to make this point salient to the participants. Notably, weight was an evaluable feature in the study by Dunn et al. This contrasts with the discussion just offered, and tentatively, we can only point to two differences. First, participants in the study by Dunn et al. were rating their perceived effort while in our study and the one of Potts et al. (2018) they needed to perform the task with the given weight. Second, the second level of weight (10 lbs) in the Dunn et al. study was higher than the heaviest buckets used here (3.2 kg or approximately 7 lbs).

With regard to the effect of cognitive task difficulty, choices did not depend on this factor in Experiment 1, but did so in Experiment 2. Thus, our expectation that multiplication would be more difficult than addition was not realized in Experiment 1. Still, an important conclusion could be reached from the fact that participants in Experiment 1 sometimes chose the cognitive task even though it had a higher error rate than the bucket task. The error rate for the bucket task was 0% but was close to 11% for the math tasks. Participants would have never chosen the math task if the sole criterion for doing so were the elimination of errors. Therefore, error elimination was not the sole basis for choosing tasks, a result observed as well by Kool et al. (2010) and Feghhi and Rosenbaum (2021). Where this leaves us is that neither error elimination nor time minimization was the sole basis for choice. Rather, all of the variables investigated here—duration of the cognitive task, its difficulty (at least in Exp. 2), and the difficulty of the physical task—influenced task choices.

How then can we account for our results? Can we do so with a single metric, as asked in the introduction of this article? Can we say there is a common currency for choosing tasks requiring less effort? We think we can.

We addressed these questions through modeling. More precisely, we asked whether the contributing factors (i.e., duration of the cognitive task, difficulty of the cognitive task, and difficulty of the physical task [i.e., bucket weight]) could be converted into a single variable. The idea was that if these factors are indeed convertible to a single subjective variable, then increasing the cognitive difficulty (e.g., from 2-digits to 4-digits in Experiment 2) should have an effect similar to increasing the duration of the 2-digit task. Similarly, increasing the duration of the cognitive task should have an effect similar to decreasing the difficulty of the physical task, that is, decreasing the bucket load.

The way we embarked on our modeling was to pursue transforms of objective duration and the other factors that would maximize the likelihood of the data. We imagined sliding three of the curves in Fig. 2 horizontally, such that all the points, plus the points along the unshifted curve, would hug a single curve. Similarly, we imagined sliding three of the curves in Fig. 3 horizontally, such that all the points, plus the points along the unshifted curve, would lie on a single curve. Finding the horizontal shifts that achieved the best fit of all the points per figure would amount to transforming the *x*-axis from objective time to a single variable referred to simply as Φ for now.

Beginning with Experiment 2 data, we defined two free parameters, denoted *k* (cognitive difficulty) and *h* (physical difficulty, i.e., bucket weight) in the following formula:

$$\Phi = \left\{ {\begin{array}{*{20}c} {duration,} & {Bucket \, weight = 3.2} & {cognitive \, difficulty = 2} \\ {duration + k,} & {Bucket \, weight = 3.2} & {cognitive \, difficulty = 4} \\ {duration + h,} & {Bucket \, weight = 0} & {cognitive \, difficulty = 2} \\ {duration + k + h} & {Bucket \, weight = 0} & {cognitive \, difficulty = 4} \\ \end{array} } \right\}$$

**Fig. 4:**
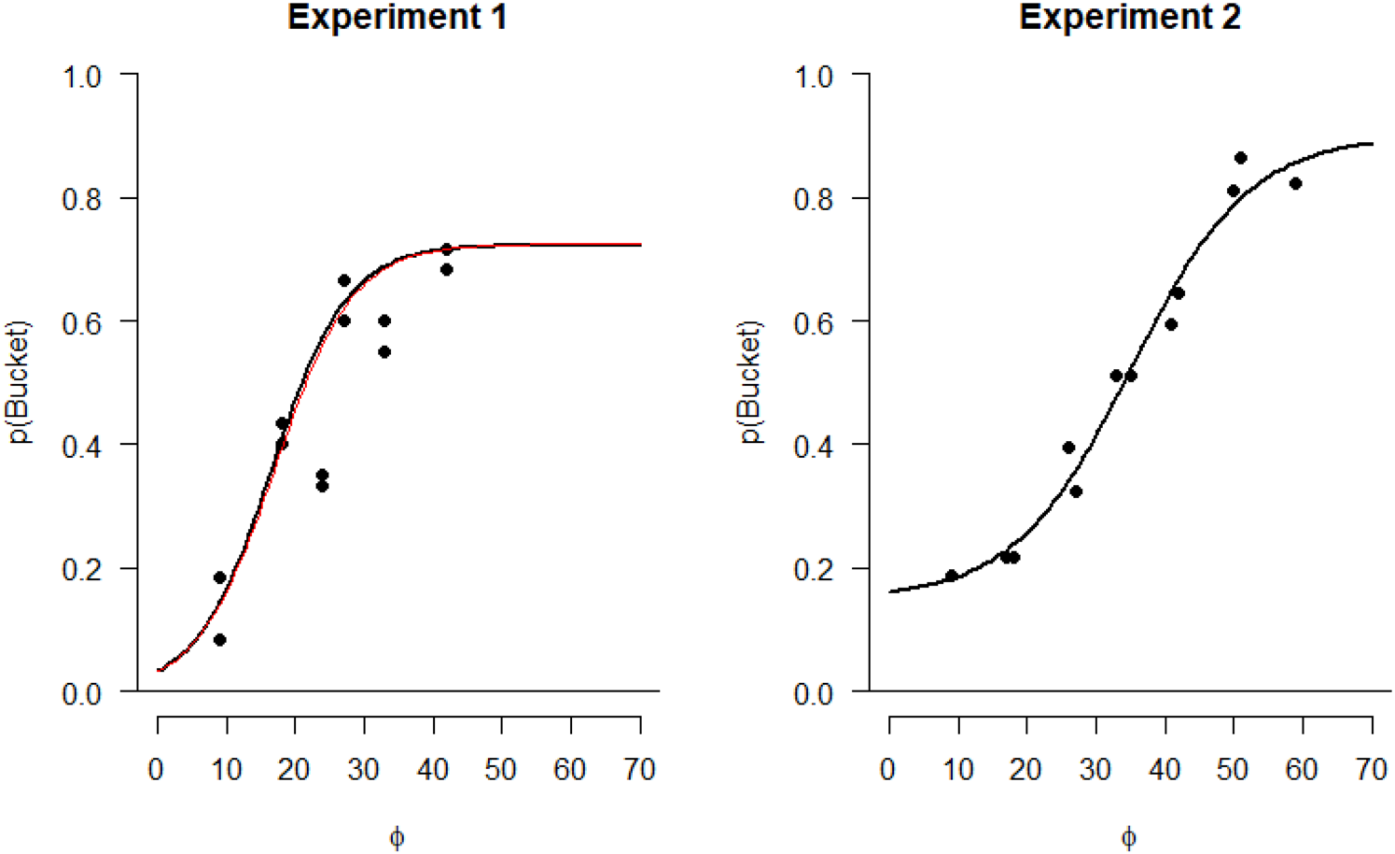
Left panel: Probability of choosing the bucket task, *p*(Bucket), in Experiment 1 as a function of a model where cognitive difficulty is the same in the addition and the multiplication task and bucket load and duration of the cognitive tasks are converted to a single variable Φ. The black line is the logistic function yielding the best fit to the data. The red line is the resulting function when using the same parameter values as obtained with the data from Experiment 2. Right panel: Probability of choosing the bucket task, *p*(Bucket), in Experiment 2. In this case, duration and difficulty of the cognitive task and bucket load were converted into a single variable Φ

For given values of *k* and *h*, we fitted a logistic regression with four parameters (upper bound, lower bound, inflection point, and steepness) to maximize the coefficient of determination, that is, ${R}^{2}=1-\frac{S{S}_{residuals}}{S{S}_{total}}$. For Experiment 2, the largest value of *R*^*2*^ was ${R}^{2}=.977,$ obtained with *h* = 8 and *k* = 24. The resulting logistic function is shown in the right panel of Fig. 4. Because we observed no effect of cognitive difficulty in Experiment 1, only one free parameter, *h,* was needed to reasonably explain choices in this experiment, and Φ would be

$$\Phi = \left\{ {\begin{array}{*{20}c} {duration,} & {Bucket \, weight = 3.2} \\ {duration + h,} & {Bucket \, weight = 0} \\ \end{array} } \right\}$$

The largest value ${R}^{2}=0.976$ was obtained for *h* = 7 in this case and the resulting logistic function is shown in the left panel of Fig. 4. When setting *h* = 8, that is, to the same value as obtained when fitting the model in Experiment 2, the fit was negligibly worse with ${R}^{2}=0.970$. The resulting logistic function is shown as the red line in the left panel of Fig. 4.

The foregoing analysis shows that all of the choice data could be modeled by expressing costs of all kinds in terms of one variable Φ. This analysis and interpretation fit well with the metacognitive framework suggested by Dunn et al. (2016) where effort is a subjective inference from multiple cues, as long as they are sufficiently salient. This inference may, but need not, covary with objective costs (e.g., in terms of [response] time). For example, Dunn and Risko (2019) pitted two tasks against each other—one with a highly salient feature but less of a time difference (stimulus rotation) and one with a clear time difference but less salient variation (task switch)—and suggested that the highly salient variant also affected choices, even though the time costs were negligible. Thus, these authors went on to suggest “to conceptualize time and demands on executive control as potential cues” (p. 8). This proposal fits well with our results that all three factors contributed to choices. The single variable Φ might therefore simply be interpreted as the effort inferred from the available and salient factors (or cues). Arguably, all factors were salient to the participants, in particular in Experiment 2, where they were required to experience both levels of cognitive task difficulty in the exposure block. We can further speculate that this rather simple model might underlie a process model for making the choices: If the participants’ subjective estimate of Φ to do a task falls below a criterion value, choose that task; otherwise, choose the other task; in case the two estimates are the same, choose at random.

A drawback of our experimental variation of cognitive task difficulty is that we cannot disentangle cognitive task difficulty and error likelihood—in fact, we operationalized less versus more difficult tasks as those inducing less versus more errors, respectively. Error likelihood has, however, been associated with the effort in a study by Dunn et al., (2019a, 2019b; but see Feghhi & Rosenbaum, 2019, 2021). Yet, Dunn et al., (2019a, 2019b) also noted a role for time judgments in effort judgment in one their experiments (Experiment 4), but identification of situations where error likelihood might play a more prominent role seems a worthwhile future direction.

Reflecting on what we have achieved here, we note that while separate studies have compared the effects of each of the three factors that we examined—cognitive difficulty, physical difficulty, and time (i.e., duration of the cognitive task)—we have shown that these factors are lawfully convertible to a single variable Φ. This variable may be interpreted as effort, but it is tempting to ask whether Φ might correspond to some other nameable construct. In keeping with the results of Potts et al. (2018), this might be subjective time. Recall that Potts et al. (2018) concluded that subjective time, over and above objective time, provided a clear account of their choice data. This interpretation also receives support from a study by Rosenbaum and Bui (2019), where it was observed that subjective time did a better job of accounting for choices than another hypothesized measure of task difficulty, task sustainability. Task sustainability was not tested in the present experiments and was only estimated by the participants in the study Rosenbaum and Bui (2019). Similarly, subjective time was not tested directly here. More work is clearly needed to test the role of this hypothesized quantity. Collecting subjective time estimates would be an obvious next step.

Despite these limitations, we feel that the present work provides a useful advance for the study of task difficulty. By asking people to choose the easier of two tasks, we found that their choices were systematic and reliable, as in previous studies. Furthermore, through modeling, we were able to map costs of different kinds onto a single variable, Φ. The functional identity of Φ remains to be determined. It could be subjective time, as just suggested, or it might be some abstract, amodal quantity, perhaps corresponding to a common code for task difficulty. The fact that several variables contributed to it is consistent with the metacognitive framework of Dunn et al. (2016). Indeed, as Dunn et al. (2016) might argue, and we agree, Φ might be interpreted as the subjective effort inferred from the salient cues of the task alternatives—in our case, duration and difficulty of the cognitive task and difficulty of the physical task.

In a related way, subjective effort has recently been described as a “common code for task difficulty”. This idea, broached by Feghhi et al. (2021), was inspired by the common coding hypothesis (Prinz, 1984, 1992; see also Hommel et al., 2001) and is meant in terms of an analogy: the traditional concept of common codes refers to the representations underlying perception and action, here it refers to the effort representation underlying different kinds of tasks. Feghhi et al. (2021) used different tasks than the ones used in the present study, but like us, they observed that choice probabilities could be well fitted with a model in which costs of different kinds mapped onto a single quantity. Feghhi et al. (2021) suggested that the quantity might be abstract and amodal, but did not attempt to argue for or against subjective time per se.

What that “true value” might be is certainly a matter for future work. The idea that subjective effort is a multi-modal or amodal representation, regardless of whether it incorporates subjective time, sustainability (Rosenbaum & Bui, 2019), error likelihood (Dunn et al., 2019a, 2019b), executive control (Kool et al., 2010) or some other feature(s) is attractive to us, for it accommodates the flexibility of task choice which is needed in everyday life. Which task is easiest in any situation additionally might depend on a wide range of situational factors. A theory of task ease or difficulty must reckon with this variability.

## Data Availability

Data is available at https://osf.io/jw98p/.

## Footnotes

1 An alternative method, asking participants to rate their perceived or anticipated effort level, has also been used and might be more suitable in some situations (e.g., Dunn et al., 2017).

2 Preferences for short reaches were also reported by Rosenbaum (2008) and Rosenbaum et al. (2011), where the alternative task was walking over some distance rather than reaching over some distance.

3 The model was $p\left(A\right)= \frac{T(B)}{T\left(B\right)+T(A)}$, where *A* and *B* were the two tasks considered per condition, and *T* was time.

4 The plausibility of this hypothesis is strengthened by the observation that psychologically experienced time differs from objective time (e.g., Grondin, 2008; Ornstein, 1969).

5 The actual time ended up being between the predetermined times (9, 18, and 27 s) and + (approx.) 1.2 s.

6 When included in an ANOVA as an additional repeated measure, location produced no significant main effect nor did the variable enter into any interactions.

7 For completeness, we report the simple paired *t* tests (two tailed) for bucket weight separately for each duration of the (4-digit) cognitive task. In this analysis, the effect of bucket weight was significant for the 18 s duration, *t*(47) = 2.55, *p* = 0.014, *d* = 0.37, but not for the 9 s duration, *t*(47) = 1.18, *p* = 0.242, *d* = 0.17, or the 27 s duration, *t*(47) = − 0.85, *p* = 0.399, *d* = − 0.12. The latter result might be attributed to a *p*(bucket) approaching ceiling. The absence of a significant difference for the 9 s duration data is unexpected, but we note that the difference is clearly into the right direction.

## Code availability

Code for the model is available at https://osf.io/jw98p/.
